# Rapid Automatized Naming and Explicit Phonological Processing in Children With Developmental Dyslexia: A Study With Portuguese-Speaking Children in Brazil

**DOI:** 10.3389/fpsyg.2020.00928

**Published:** 2020-05-27

**Authors:** Patrícia Botelho da Silva, Pascale M. J. Engel de Abreu, Paulo Guirro Laurence, Maria Ângela Nogueira Nico, Luiz Gustavo Varejão Simi, Rute C. Tomás, Elizeu Coutinho Macedo

**Affiliations:** ^1^Social and Cognitive Neuroscience Laboratory, Developmental Disorders Program, Center for Health and Biological Science, Mackenzie Presbyterian University, São Paulo, Brazil; ^2^Institute for Research on Multilingualism, University of Luxembourg, Belval, Luxembourg; ^3^Brazilian Dyslexia Association (ABD, Associação Brazileira de Dislexia), São Paulo, Brazil

**Keywords:** developmental dyslexia, rapid automatized naming, explicit phonological processing, Brazilian Portuguese, classification and regression tree analysis

## Abstract

Many studies have shown that children with reading difficulties present deficits in rapid automatized naming (RAN) and phonological awareness skills. The aim of this study was to examine RAN and explicit phonological processing in Brazilian Portuguese–speaking children with developmental dyslexia and to explore the ability of RAN to discriminate between children with and without dyslexia. Participants were 30 children with a clinical diagnosis of dyslexia established by the Brazilian Dyslexia Association and 30 children with typical development. Children were aged between 7 and 12, and groups were matched for chronological age and sex. They completed a battery of tests that are commonly used in Brazil for diagnosing dyslexia, consisting of the Wechsler Intelligence Test for Children (WISC-IV) as well as tests of single word and non-word reading, RAN, and the profile of phonological abilities test. Results indicate that the cognitive profile of this group of children, with a clinical diagnosis of dyslexia, showed preserved skills in the four subscales of the WISC-IV (verbal comprehension, perceptual reasoning, working memory, and processing speed) and on the profile of phonological abilities test. Groups significantly differed on the reading tests (word and non-word) and RAN measures, with medium to large effect sizes for RAN. Classification and regression tree analysis revealed that RAN was a good predictor for dyslexia diagnosis, with an overall classification accuracy rate of 88.33%.

## Introduction

Dyslexia is a neurodevelopmental disorder, affecting between 5 and 10% of the general population (Zeffiro and Eden, 2000). Developmental dyslexia is generally characterized by a specific and persistent difficulty in the acquisition of literacy that cannot be explained by apparent deficits in other cognitive abilities or insufficient educational opportunities (Caravolas et al., 2012). According to the diagnostic criteria set forth in diagnostic guides such as the *DSM-5* (*Diagnostic and Statistical Manual of Mental Disorders*, American Psychiatric Association [APA], 2014), dyslexia is classified as a “specific learning disorder” with impairment in reading. Symptoms include difficulties in reading accurately and fluently that interfere with scholastic achievement and everyday life and that are inconsistent with the child’s chronological age, intellectual abilities, and educational opportunities (American Psychiatric Association [APA], 2014). In addition, problems with spelling and writing are often observed that can be more persistent and severe than the reading problems (Maughan et al., 2009).

Children with dyslexia primarily present difficulties that affect word-level decoding skills, and they struggle to master the relationship between spelling patterns and the pronunciation of words (Handler et al., 2011; Snowling and Hulme, 2012). Decoding is commonly assessed by tests of word identification (i.e., reading aloud single words) and reading aloud non-words, which taps more directly into phonological decoding skills. Although dyslexia is used as a categorical diagnosis, it has been suggested that dyslexia is dimensional in nature and presents continuities with other language disorders (Snowling and Hulme, 2012). As such, dyslexia has been reconceptualized as a language disorder in which phonological language skills are deficient and that can present itself in different ways during the course of development (Catts, 1991; Bishop and Snowling, 2004). Decoding difficulties may also lead to reduced exposure to print, which in turn weakens vocabulary development, and writing expression. It is possible that individuals with dyslexia can achieve adequate word reading accuracy; however, they generally present persistent reading fluency deficits and comprehension difficulties due to slow and non-automatized reading (Handler et al., 2011; Norton and Wolf, 2012; Peterson and Pennington, 2012).

Cognitive performance measures reveal that individuals with dyslexia can present deficits in a broad range of tasks including impairments in phonological, visuospatial, attentional, working memory, and executive function measures (Menghini et al., 2010; Zoubrinetzky et al., 2014; Tamboer et al., 2016). Although different subtypes of dyslexia are discussed in the literature (Giofrè et al., 2019), there is a general consensus that children with dyslexia present weaknesses in phonological processing skills (Snowling, 2001; Ramus et al., 2003; Vellutino et al., 2004; Ziegler and Goswami, 2005). According to the most widely accepted view in the field (see Hulme and Snowling, 2009, for a review), decoding deficits seen in dyslexia arise from poorly specified phonological representations (the phonological representations hypothesis). According to this theory, problems in learning to read and difficulties in phonological processing tasks reflect a basic deficit in how phonological information is represented in the brain. Results from a large number of studies across languages have shown that individuals with dyslexia have difficulties with a wide range of implicit and explicit phonological processing tasks (Ramus et al., 2003; Vellutino et al., 2004; McBride-Chang et al., 2008). It has been argued that implicit phonological processing tasks engage phonological processing automatically without any explicit reflection on it, such as tasks of rapid automatized naming (RAN) or verbal short-term memory (Melby-Lervåg et al., 2012). Explicit phonological processing tasks, on the other hand, require the conscious reflection and manipulation of speech sounds in words. These tasks are often referred to as phonological awareness tests. Deficits in phonological awareness and RAN tasks have been consistently reported in children with dyslexia, and it has been suggested that difficulties in such tasks are among the characteristic features of dyslexia (Pennington and Lefly, 2001; Landerl et al., 2013; Araújo and Faísca, 2019).

Phonological awareness has been defined as the ability to recognize, discriminate, and manipulate speech sounds of spoken words. It is best understood as a complex construct composed of separate subskills (Muter et al., 2004; Melby-Lervåg et al., 2012). Its development is believed to follow a hierarchical pattern, progressing from larger sound units, such as syllables, to smaller units, such as phonemes (Stanovich, 1992). Phonological awareness can be assessed by a wide variety of tasks. These tasks often vary greatly from each other in terms of the size of the phonological unit that needs to be analyzed or manipulated as well as the type of judgment involved in the task (Vloedgraven and Verhoeven, 2007; Melby-Lervåg et al., 2012). Across orthographies, phonological awareness has been identified as a strong predictor of success in learning to read (Capovilla and Capovilla, 2003; Anthony and Francis, 2005; Hogan et al., 2005; Fricke et al., 2008; Engel de Abreu and Gathercole, 2012; Melby-Lervåg et al., 2012; Rothou et al., 2013). Phoneme-level skills tend to be a better predictor of early reading skills than phonological awareness tasks involving larger linguistic unit size (Caravolas et al., 2005). Several studies have also shown that training phonemic awareness (ideally in combination with letter sound knowledge) can help to improve reading skills in children (Hatcher et al., 2004; Bowyer-Crane et al., 2008; National Institute for Literacy, 2008). Taken together, evidence from longitudinal and intervention studies indicates that phonemic awareness is likely to exert a causal influence on reading development and is reciprocally linked to literacy experience (Catts et al., 2001; Melby-Lervåg et al., 2012).

An abundance of research has shown that children with dyslexia present significant impairments in tasks of phonological awareness in general and phonemic awareness in particular (see Melby-Lervåg et al., 2012 for a review). This is also a consistent finding reported in studies from Brazil with children learning to read in Portuguese (Capovilla et al., 2001; Dourado et al., 2005; Capellini et al., 2007, 2008; Lima et al., 2008; Deuschle and Cechella, 2009; Germano et al., 2009). In their systematic meta-analytic review, Melby-Lervåg et al. (2012) showed that, independent of orthography, children with dyslexia had a large deficit in phonemic awareness in comparison to their typically developing peers, and this deficit was noticeably larger than differences between groups in rime awareness.

Another, less well-understood predictor of variations in reading development is RAN. RAN deficits are also frequently observed in children with, or at risk of, reading difficulties (see Araújo et al., 2015; Araújo and Faísca, 2019, for a review). RAN tasks require naming familiar items (objects, colors, letters, or digits) that are visually presented as fast as possible. These simple tasks tap different cognitive skills including speed of processing, visual and integration skills, executive function, as well as access to phonological representations (Alves et al., 2016). Concurrent and longitudinal studies have shown that RAN correlates with reading accuracy and early reading fluency (Norton and Wolf, 2012; Araújo et al., 2015). RAN tasks can be separated according to the stimuli used. A common distinction is between RAN tasks that use alphanumeric (letters and digits) and non-alphanumeric (objects and colors) stimuli. Non-alphanumeric RAN has been shown to predict reading for children who have not entered school yet or have had insufficient experience with letters or numbers. The relationship between RAN and reading is therefore not just a consequence of differences in digit or letter knowledge (Lervåg and Hulme, 2009). Alphanumeric RAN has been found to be a stronger correlate of reading in school-aged children who had started formal literacy instruction (Araújo et al., 2015). It has been argued by some that RAN is the predominant predictor of individual differences in reading in consistent orthographies (Mayringer and Wimmer, 2000), but others have shown different results (Kirby et al., 2010). Findings from a large-scale longitudinal study including different European orthographies showed that both RAN and phonological awareness were reliable predictors of reading skills, with equal relative importance (Caravolas et al., 2012). The study concludes that the underlying cognitive processes involved in learning to read in consistent and less consistent orthographies are identical. Studies with extreme groups have shown that individuals with dyslexia performed slower and made more mistakes on RAN tasks when compared to typical readers (Araújo and Faísca, 2019). This pattern of results persists throughout development and is relatively stable even in compensated readers with dyslexia or high-functioning dyslexics. RAN deficits are, however, not specific to dyslexia but can also be observed in other neurodevelopmental disorders such as attention-deficit/hyperactivity disorder.

The underlying mechanism driving the relationship between RAN and reading is currently not well understood, and there are a number of competing theories (Jones et al., 2013; Protopapas et al., 2013). According to Wimmer et al. (2000), the observed relationship is due to RAN and reading both relying on the speed of phonological retrieval from long-term memory. A related hypothesis, put forward by Lervåg and Hulme (2009), is that RAN and reading tap the same brain systems involved in mapping between visual and phonological codes. Intervention studies that attempt to improve rapid naming are scarce and have generated mixed findings. Whereas some studies indicate that training rapid naming has no reliable effect on RAN or reading (Kirby et al., 2010), other studies have found that training RAN can have a positive effect on word-level reading skills (Stappen and Reybroeck, 2018).

In relation to dyslexia, one view is that phonological awareness and RAN are both deficient because they are related subcomponents tapping into phonological abilities that are impaired in dyslexia (Wagner and Torgesen, 1987; Melby-Lervåg et al., 2012). Others have argued that RAN should be considered an entirely different cause of reading difficulties and that naming speed represents a second core deficit in developmental dyslexia. In their influential “double-deficit hypothesis,” Wolf and Bowers (1999) propose that phonological awareness and RAN are two independent sources of reading impairment. According to this theory, three major types of impaired readers can be identified (Wolf and Bowers, 1999; Norton and Wolf, 2012): children with a single deficit in phonological awareness (the phonological-deficit subtype), children with a single deficit in RAN (the RAN-deficit subtype), and children with a double deficit (phonological awareness and RAN-deficit subtype). The broadest and most persistent reading difficulties are experienced by the latter subtype (Wolf and Bowers, 1999; Lovett et al., 2000; Manis et al., 2000; Kirby et al., 2003, 2010). It has been suggested that children with a single-deficit profile present difficulties in specific components of reading. Whereas RAN deficits might primarily affect reading fluency, impairments in phonological awareness have been more extensively linked to spelling difficulties (Wimmer et al., 2000; Torppa et al., 2013). A number of studies have identified such profiles of readers across different alphabetic writing systems (Wolf et al., 2002; Escribano, 2007; Papadopoulos et al., 2009; Araújo et al., 2010; Torppa et al., 2013). In a study with Portuguese-speaking children with dyslexia, Araújo et al. (2010) managed to find the predicted subtypes. The authors suggest that their findings might be related to the more important role RAN skills might play in the Portuguese orthography than in less consistent orthographies such as English (see also Seymour et al., 2003). However, others have failed to find impaired RAN without affected phonological awareness skills in developmental dyslexia (Badian, 1997; Vaessen et al., 2009), and the literature relating to the double-deficit hypothesis remains controversial (Ackerman et al., 2001; Schatschneider et al., 2002; Vukovic and Siegel, 2006; Vaessen et al., 2009). Inconsistencies in findings might be related to the fact that different studies use different measures of RAN, phonological awareness, and reading. Yet, there appears to be a general consensus in the field that dyslexia is a heterogeneous disorder and that poor reading performance can be associated with different cognitive profiles (Wolf and Bowers, 1999; Pennington, 2006; Snowling, 2008; Peterson and Pennington, 2012; Norton et al., 2014). Severity of the underlying deficits, general processing resources, as well as reading experience are all factors that can have an effect on how reading difficulties manifest themselves in individual children (Griffiths and Snowling, 2002).

Although RAN and phonological awareness have been extensively studied in children learning to read in English, considerably fewer studies have focused on other languages and literacy instruction contexts. Findings from a recent study in Brazil have shown that deficits in both phonological awareness and RAN were associated with persistent reading difficulties in a group of children showing little progress in learning to read in the early school years (Michalick-Triginelli and Cardoso-Martins, 2015). In the current study, we explored RAN and explicit phonological processing in a group of children with a clinical diagnosis of dyslexia from Brazil who were learning to read in Portuguese. Compared to English, the Portuguese orthography is more consistent and falls in an intermediate position on the transparency–opacity continuum (Scliar-Cabral, 2003; Seymour et al., 2003). In Portuguese orthography, vocalic grapheme–phoneme correspondences are not very regular. Consonant grapheme–phoneme correspondences are more regular but also exhibit some complexity. To read irregular words, children need to have acquired specific strategies such as whole word reading (Cardoso-Martins, 2005). Studies exploring reading acquisition and dyslexia have shown that the profile of readers of Portuguese is different from that described for shallow orthographies and deep orthographies, consistent with the view that Portuguese is an orthography of intermediate depth (Seymour et al., 2003; Sucena et al., 2009). In Brazil, formal literacy instruction starts in the first grade when children are 6, and reading is expected to be accurate and fluent by the end of the second grade (BRASIL, 2018). However, national and international studies have shown that many children in Brazil can present difficulties in acquiring basic literacy skills (BRASIL, 2019; OECD Programme for International Student Assessment [PISA], 2019). A major challenge in Brazil is to differentiate children with dyslexia from children with reading difficulties because of insufficient reading experience or instruction. Studies exploring the predictive efficiency of RAN tests for dyslexia likelihood are therefore relevant for both theory and practice.

A major objective of this study was to investigate RAN and explicit phonological processing in Brazilian Portuguese–speaking children with developmental dyslexia. A specific interest was to determine whether children with dyslexia in Brazil would manifest deficits in rapid naming and to explore the predictive ability of RAN to discriminate between children with and without dyslexia. We therefore investigated the performance of children with and without dyslexia on reading measures, RAN tasks, the profile of phonological abilities test, and the Wechsler Intelligence Scale for Children. The profile of phonological abilities test (*Perfil de Habilidades Fonológicas*, PHF, Carvalho et al., 1998) is a commonly used instrument in Brazil for the screening and diagnosis of reading difficulties in children. It is a relatively broad assessment of phonological processes in Portuguese, combining tasks of phonological awareness (at different phonological unit sizes) as well as analyses at the sentence level and articulation. Based on the phonological representations account of dyslexia and earlier studies in the field (Hulme and Snowling, 2009), we expected that children with dyslexia would be impaired in explicit phonological processing. Following the view that RAN taps a fundamental mechanism that places constraints on reading development (Lervåg and Hulme, 2009), we also expected that children with dyslexia would present deficits on RAN tasks and that RAN and explicit phonological processing would predict dyslexia likelihood in children from Brazil.

## Materials and Methods

### Participants

A total of 60 participants aged 7–12 years took part in this study: 30 children [25 (83.3%) males; mean age: 9.70 years, *SD* = 1.51 months] with a clinical diagnosis of dyslexia and 30 children [25 (83.3%) males; mean age: 9.77 years, *SD* = 1.54 months] without a clinical diagnosis who served as a control group. Children in the clinical group were individually matched with children from the control group on chronological age and sex. All participants were monolingual Portuguese speakers with normal or corrected vision and hearing. The typical readers all attended private schools in the city of São Paulo. Only typically developing children with no reported difficulties in reading and spelling (based on school records and teacher reports) were included in the study. Children in the clinical group came from different states in Brazil, and 93% of the children frequented private schools. The dyslexia diagnosis had been established by the Brazilian Dyslexia Association (*Associação Brasileira de Dislexia*, ABD) based on *DSM* criteria. The precise diagnostic criteria used were the following: reading of at least 1.5 years behind the chronological age, scores on word-level decoding of at least one standard deviation below the mean, and no intellectual disability. Children with an additional diagnosis of a neurodevelopmental disorder, hearing or visual impairments, or a standard score below 85 on the Wechsler Intelligence Test for Children—Fourth Edition (WISC-IV) were not included in the study. Informed consent to participate in the research was obtained from participating parents and children. Ethical approval for the study was granted by the Mackenzie Presbyterian University (UPM) Research Ethics Committee (CAAE: 80902017.8.0000.0084) in accordance with UPM requirements.

### Procedure

Children in the clinical group had been assessed by a multidisciplinary team (including neuropsychologists, psycho-pedagogues, speech and language therapists, and medical doctors) of ABD at the Specialized Center for Learning Disorders (CEDA, *Centro Especializado em Distúrbios de Aprendizagem*) in São Paulo city. Each child completed a comprehensive battery of tests as part of the dyslexia diagnostic assessment. The evaluation also included a detailed medical and developmental history. Children had been referred to ABD by parents or educators and were tested between 2015 and 2019. The research team from UPM was granted access to clinical records, and the data on the measures of interest were made available for the purpose of this research project (As part of the initial assessment at ABD, parents were given the option to make their child’s data available for research undertaken at UPM). Children in the control group were recruited from two private schools in São Paulo city. School records and teacher reports of individual children were examined, and only children with no known developmental disorder or academic difficulties were invited to take part in the study. All the children in the control group were individually assessed by the first author, who is a licensed psychologist, and by trained graduate students in psychology. Testing took place in a quiet area of the schools in three sessions lasting approximately 1 h each on different school days. Tests were administered in a fixed sequence to all participants.

### Measures

#### Cognitive Ability Measure

Children completed the Brazilian version of the *WISC-IV* (Wechsler, 2013, Brazilian adaptation by Rueda et al., 2013) as a test of intellectual ability and cognitive processing. The WISC-IV has 15 subtests (of which 10 are core subtests) that can be clustered into the following four index scores: Verbal Comprehension Index (VCI), Perceptual Reasoning Index (PRI), Working Memory Index (WMI), and Processing Speed Index (PSI). In addition, the test gives a full-scale IQ score. The test was administered and scored according to the manual. Standard scores (full-scale and index scores: *M* = 100, *SD* = 15; subtest scores: *M* = 10, *SD* = 3) were used in the statistical analyses. Test–retest reliability of the different WISC-IV indexes reported from the manual (Wechsler, 2013, Brazilian version) ranges from good to excellent (between 0.86 and 0.93).

#### Rapid Automatized Naming

Children were administered a Portuguese version of the RAN test developed by Wolf and Denckla (2005) that requires children to sequentially name a list of items as fast as possible. The test includes four single-category naming tasks [colors (red, yellow, black, blue, green), digits (two, six, four, seven, nine), objects (book, chair, dog, hand, star), and lowercase letters (a, o, s, d, p)]. It is composed of four A3-size stimulus cards containing 50 stimuli each (with each item repeated 10 times) presented in rows of 10 items and with no adjacent item repetitions. Children are asked to name all the stimuli as accurately and as fast as possible. For the letter naming subtest, children are instructed to say letter names. The four RAN subtasks were administered in the following fixed sequence to all the children: colors, digits, objects, and letters. The time taken to name the 50 stimuli on each subtest was recorded in seconds. The numbers of naming errors and self-corrections were also recorded for each RAN subtest. Self-corrections were scored as correct responses. The total time score was the time in seconds needed to name all the items. In addition to total time scores on the single-category RAN subtests, three total time composite scores were computed: a total score from the four RAN subtests, an alphanumeric score from the number and letter naming subtests, and a non-alphanumeric score from the color and object naming subtests.

#### Explicit Phonological Processing

Children completed the *profile of phonological abilities test* (PHF, Carvalho et al., 1998). This standardized test was designed to assess different aspects of phonological processing in Brazilian children aged 5–10 years. The PHF comprises nine subtests: syllabic analyses, syllable and phoneme blending, segmentation of sentences (into words) and words (into syllables), syllable and phoneme deletion, syllable and phoneme substitution, rime detection, sequential memory for rime, syllabic reversal, and articulatory image. Each subtest contains two training items and four test items. Correct answers receive a score of 1 or 2, depending on the subtest, with a total maximum score of 76 on the entire test. The total score was used as a dependent variable in all the analyses (sub-scores were not available for analyses for the group of children with dyslexia). Raw scores were used because the test has not been standardized on children who are older than 10 years. Reliability was established on the sample of 30 children in the control group. The test had good internal consistency, with a McDonald’s omega of 0.98 (McDonald, 1999).

#### Word-Level Reading

Children completed a *single word reading* and a *non-word reading* test that were developed by ABD (unpublished). In each case, children are required to read aloud lists of words or non-words quickly and accurately. The single word reading tests consists of 94 items, and the non-word reading test consists of 96 items. Items of increasing complexity are presented in six columns on a standard A4-size sheet. Children are asked to read all the words/non-words. An item is considered as correct if the whole word/non-word is read correctly in Portuguese. No credit is given if children read individual phonemes. The word reading test contains unrelated words that progress in difficulty, beginning with disyllabic words. In total, the test contains 72 disyllabic, 20 trisyllabic, and 2 quadrisyllabic words. Non-words in the non-word reading test are of two general types: non-words that are close to real words in Portuguese (derived by changing one or more letters in a real word, such as *plumão* derived from *pulmão*) and non-words that are unrelated to real words (that could not be easily linked to any real word such as *flaus* or *afe*). The test contains 18 monosyllabic, 54 disyllabic, 22 trisyllabic, and 2 quadrisyllabic non-words. For each test, the dependent variables used for analyses were reading accuracy (total number of items read correctly), reading rate in minutes (total time needed to read all the items), and reading efficiency (accuracy/rate). As no norms are available on these tests, raw scores were used in all the analyses.

## Results

Descriptive and inferential statistics were computed with IBM SPSS Statistics for Windows, version 22.0 (IBM Corp. Released, 2013, Armonk, NY, United States). The level of significance for the interpretation of analyses was set at or below 5%. Student’s *t*-tests were performed to compare the performance of children in the two groups on most measures. To correct for the effect of multiple tests on the likelihood of a type I error, *p*-Values were adjusted to control for false discovery rates using the Benjamini and Hochberg (BH) method (Benjamini and Hochberg, 1995) with R (R Core Team, 2014). Following Jafari and Ansari-Pour (2019), the BH procedure was applied to the complete set of tests as measures were related. The variables manifested reasonable univariate normality, and the measures did not present floor or ceiling effects (all means were at least 1 *SD* from the maximum and minimum scores). Levene’s test showed that the variances for some measures were not equal. In these cases, Welsh’s *t*-tests were used.

Descriptive statistics for the WISC-IV measures are provided in Table 1. Children with dyslexia did not differ significantly from the controls on the majority of the cognitive ability scores. Significant differences emerged, however, on the verbal subtest scores similarities, *t*(58) = 2.34, *p* < 0.05, *d* = 0.60, and information, *t*(58) = 2.35, *p* < 0.05, *d* = 0.61, as well as on the working memory subtest score arithmetic, *t*(58) = 2.35, *p* < 0.05, *d* = 0.61. In each case, children in the control group outperformed children in the dyslexia group, and effect sizes were medium in magnitude. Results on the RAN tests, the explicit phonological processing measure, and the reading measures are presented in Table 2. As expected, considering the inclusion criteria, children with dyslexia performed significantly less well than their typically developing peers on the word-level reading measures. Effect sizes were large, with Cohen *d*s ranging from 1.00 to 1.92. It is noteworthy that children in the control group manifested comparable performance in word and non-word reading accuracy (*M*_*word reading*_ = 91.43; *M*_*non–word reading*_ = 91.27), whereas children with dyslexia had higher scores in word reading accuracy as compared to non-word reading (*M*_*word reading*_ = 72.47; *M*_*non–word reading*_ = 64.07). No significant difference between the groups emerged on the explicit phonological processing task (*d* = 0.20). Results on the RAN tasks are clear. Children in the dyslexia group were significantly slower in RAN and made significantly fewer self-corrections than children in the control group. Effect sizes ranged from medium to large on the total time measures (*d*s between 0.66 and 0.91). The largest effect size emerged on letter naming RAN (*d* = 0.91). Notably, children with dyslexia made significantly more mistakes in rapid letter naming, *t*(58) = 2.46, *p* < 0.05, *d* = 0.64, but not in the other RAN tests (using different stimuli).

**TABLE 1:** Mean standard scores (*SD*) with minimum and maximum scores on the WISC-IV measures according to group, with significance tests and effect sizes.

	Dyslexia group			Control group				Significance tests		
	*N* = 30			*N* = 30				with effect sizes		
Index and subtest scores	Mean (*SD*)	Min	Max	Mean (*SD*)	Min	Max	*t*	*p*	*q*	*d*
Total score	115.23 (11.75)	97	139	116.20 (10.40)	95	139	–0.34	0.74	0.84	0.09
Verbal comprehension	114.17 (14.87)	61	146	119.07 (12.39)	88	148	–1.39	0.17	0.27	0.36
Similarities	12.73 (2.88)	6	19	14.33 (2.40)	10	19	–2.34	0.02	0.04	0.60
Vocabulary	12.83 (2.52)	9	19	13.13 (2.81)	6	18	–0.44	0.67	0.79	0.11
Comprehension	12.47 (1.78)	8	17	12.50 (2.22)	7	17	–0.06	0.95	0.96	0.02
Information	11.70 (3.37)	5	19	13.53 (2.65)	8	19	–2.35	0.02	0.04	0.61
Reasoning with words	12.83 (2.99)	8	19	12.37 (3.14)	6	19	0.58	0.57	0.71	0.15
Perceptual reasoning	117.77 (12.59)	94	140	115.93 (12.89)	90	138	0.56	0.58	0.71	0.14
Block design	12.73 (2.83)	8	18	12.97 (2.71)	6	17	–0.33	0.75	0.84	0.08
Picture concepts	13.00 (2.61)	8	17	11.93 (2.57)	7	16	1.58	0.12	0.20	0.41
Matrix reasoning	13.14 (2.53)	8	17	13.10 (2.80)	7	18	0.06	0.96	0.96	0.01
Picture completion	12.45 (2.38)	9	17	11.13 (2.46)	6	16	2.08	0.04	0.08	0.54
Working memory	102.70 (12.38)	77	135	104.73 (11.46)	83	132	–0.66	0.51	0.68	0.17
Digit span	10.10 (2.78)	5	19	9.97 (2.43)	4	16	0.20	0.84	0.91	0.05
Letter–number sequence	10.83 (1.89)	7	15	11.63 (2.31)	8	18	–1.46	0.15	0.24	0.38
Arithmetic	10.83 (2.12)	8	16	12.30 (2.69)	8	18	–2.35	0.02	0.04	0.61
Processing speed	105.50 (10.57)	83	126	106.03 (14.05)	71	144	–0.66	0.87	0.91	0.04
Coding	10.43 (1.91)	5	15	10.90 (2.91)	2	16	–0.73	0.47	0.65	0.19
Symbol search	11.47 (2.01)	8	16	10.96 (2.20)	6	16	0.91	0.37	0.54	0.24
Cancelation	10.45 (2.72)	5	15	9.27 (2.88)	3	15	1.62	0.11	0.20	0.42

*Full-scale and index scores are standard scores with a mean = 100 (*SD* = 15); subtest scores are standard scores with a mean = 10 (*SD* = 3); *q*-Values are Benjamini–Hochberg adjusted; all the effect sizes are Cohen *d*. WISC-IV = Wechsler Intelligence Test for Children-Fourth Edition.*

**TABLE 2:** Mean raw scores (*SD*) with minimum and maximum scores on RAN, explicit phonological processing, and reading measures according to group, with significance tests and effect sizes.

	Dyslexia group			Control group				Significance tests		
	*N* = 30			*N* = 30				with effect sizes		
	Mean (*SD*)	Min	Max	Mean (*SD*)	Min	Max	*t*	*p*	*q*	*d*
**RAN**										
Total time (in seconds)										
Color	58.73 (22.12)	37	117	47.17 (11.59)	34	92	2.54	0.01^*a*^	0.03	0.66
Object	50.57 (15.08)	19	95	42.43 (8.11)	31	65	2.60	0.01^*a*^	0.03	0.67
Letters	43.07 (22.74)	24	143	27.73 (7.10)	20	54	3.53	0.00^*a*^	0.00	0.91
Numbers	37.87 (16.71)	23	108	27.63 (5.93)	19	39	3.16	0.00^*a*^	0.01	0.82
Non-alphanumeric	109.30 (34.21)	74	209	89.60 (18.56)	67	155	2.77	0.01^*a*^	0.02	0.72
Alphanumeric	80.93 (39.09)	48	251	55.37 (11.86)	40	92	3.43	0.00^*a*^	0.00	0.89
Total	190.23 (68.39)	132	460	144.97 (25.05)	111	208	3.40	0.00^*a*^	0.00	0.88
Number of errors										
Color	0.47 (0.76)	0	3	0.43 (0.73)	0	2	0.17	0.86	0.91	0.05
Object	0.33 (0.76)	0	3	0.23 (0.50)	0	2	0.60	0.55	0.71	0.16
Letters	2.20 (3.56)	0	15	0.50 (1.25)	0	6	2.46	0.02^*a*^	0.04	0.64
Numbers	0.37 (0.85)	0	4	0.20 (0.48)	0	2	0.93	0.33	0.51	0.24
Number of self-corrections										
Color	0.07 (0.25)	0	1	0.70 (0.79)	0	3	–4.16	0.00^*a*^	0.00	1.07
Object	0.10 (0.40)	0	2	0.63 (0.72)	0	2	–3.55	0.00^*a*^	0.00	0.92
Letters	0.03 (0.18)	0	1	0.40 (0.72)	0	2	–2.69	0.01^*a*^	0.02	0.70
Numbers	0.07 (0.25)	0	1	0.47 (0.63)	0	2	–3.23	0.00^*a*^	0.01	0.83
Explicit phonological processing										
PHF test (76)	61.50 (7.14)	40	71	62.83 (6.44)	49	74	–0.76	0.45	0.64	0.20
**Reading**										
Word reading										
Accuracy (94)	72.47 (19.06)	7	90	91.43 (2.29)	85	94	–5.41	0.00^*a*^	0.00	1.40
Rate (minutes)	3.18 (1.79)	1	11	1.39 (0.70)	0,5	4,3	5.12	0.00^*a*^	0.00	1.32
Efficiency	29.10 (16.44)	3	80	78.46 (34.22)	20	170	–7.12	0.00^*a*^	0.00	1.84
Non-word reading										
Accuracy (96)	64.07 (19.81)	8	83	91.27 (3.15)	84	96	–7.43	0.00^*a*^	0.00	1.92
Rate (minutes)	3.05 (1.02)	1	5	2.11 (0.87)	1,2	5,2	3.82	0.00	0.00	1.00
Efficiency	23.91 (11.79)	2	57	49.38 (17.17)	16	79	–6.66	0.00^*a*^	0.00	1.73

*() maximum raw scores; PHF, profile of phonological abilities test (*Perfil de Habilidades Fonológicas*); RAN, rapid automatized naming. ^*a*^Welsh’s *t*-test. *q*-Values are Benjamini–Hochberg adjusted; effect sizes are Cohen *d*.*

To explore the predictive ability of RAN scores for dyslexia diagnosis, classification and regression tree (CART) analysis was performed. The CART method is based on recursive partitioning analysis and does not require parametric assumptions. It is based on decision tree algorithms and involves the separation of different values of classification variables through progressive binary splits (Breiman et al., 2017). It is considered better suited for generating clinical decision rules than logistic regression (Lewis, 2004). Furthermore, the CART method has been shown in previous research to provide accurate group membership when groups consist of multiple latent subgroups. Such is the case here: within the group of children with dyslexia, there exist differentiated levels of actual reading deficit severity. In a recent simulation study, more traditional methods for classification, such as linear discriminant analysis or logistic regression, have been shown not to perform well in the presence of known group mixtures, and CART was identified as the method that provided the most accurate predictions in such situation (Holmes Finch et al., 2014).

**FIGURE 1:**
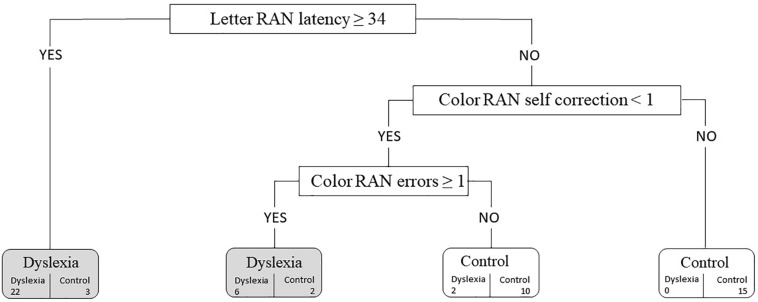
The decision tree generated by the classification and regression tree (CART) analysis along with the dyslexia diagnosis data for each child node of the tree. Selected variables with cutoff values are represented in the boxes. Numbers at the bottom of the boxes with rounded corners represent the true number of individuals in each group.

Classification and regression tree analysis was conducted in R using RPART (Recursive Partitioning and Regression Trees, Therneau and Atkinson, 2019). The stopping rule for nodes was set at <20 observations, and the complexity parameter was set at 0.01. Surrogate splits were used to deal with missing data. The 15 RAN measures and the explicit phonological processing measure were introduced into the model. Of the 16 variables evaluated, the CART method identified letter RAN total time, color RAN self-corrections, and color RAN errors as predictors of group membership. Figure 1 depicts the decision tree generated by the CART analysis along with the dyslexia diagnosis data for each child node of the tree. The first variable selected for splitting was letter RAN total time. Children with values of 34 or above were classified as children with dyslexia. The group that had letter RAN total time values below 34 was further split according to number of self-corrections on color RAN. Children who had made one or more self-corrections were classified as children without dyslexia. Those who had made no self-correction were further differentiated by number of errors on color RAN. Participants who had made one or more errors were classified as children with dyslexia. The accuracy of the model was 88.33%. According to Swets (1988), the model is of moderate diagnostic accuracy (poor: 50–70%; moderate: 70–90%; high: 90–100%). Seven participants had been misclassified: two children with dyslexia had been predicted to be in the control group (sensitivity of 93.33%), and five children from the control group had been predicted to have a dyslexia diagnosis (specificity of 83.33%). To explore how accurately the predictive model would perform in an independent data set, we used the method of cross-validation in R using RPART (Tattar, 2017). Three new models were produced: for the first model, accuracy was 26.67% (44 participants were misclassified); for the second model, accuracy was 75.00% (15 participants were misclassified); and for the third model, accuracy was 75.00% (15 participants were misclassified). Results from this cross-validation analysis indicate that the generalizability of the model to an independent data set might be limited.

## Discussion

This study examined the cognitive profile associated with developmental dyslexia in a sample of 60 children from Brazil and explored whether RAN is relevant for predicting dyslexia likelihood. The research contributes to existing literature on RAN and explicit phonological processing in dyslexia by exploring these processes in children growing up in Brazil. In contrast to research with children learning to read in English, relatively little research exists on this topic in readers of Brazilian Portuguese and in children with a clinical diagnosis of dyslexia. Measures included established predictors of reading (RAN and explicit phonological processing), the WISC-IV, and traditional measures of word-level reading skills. We compared the performance of children with dyslexia to a chronological age-matched control group of typical readers, and we used CART analysis to identify potential variables as significant predictors of dyslexia diagnosis. This statistical technique has proven to be an effective classification tool especially in situations where classification groups are not homogeneous (Holmes Finch et al., 2014).

Findings suggest that dyslexia is associated with deficits in word and non-word reading. Notably, differences between the groups were more pronounced for non-word than for word reading accuracy. This result is in line with previous research from other language-learning contexts (Snowling, 2001; Ramus et al., 2003; Vellutino et al., 2004; Ziegler and Goswami, 2005; Pennington, 2009) and extends it to children from Brazil who are learning to read in Portuguese. There are strands of evidence suggesting that a likely source of deficit in non-word reading is poorly specified phonological representations (see Hulme and Snowling, 2009 for a review). An unexpected and surprising finding was that groups did not differ on the measure of explicit phonological processing. This appears to contradict the established theory that the proximal cause of developmental dyslexia is a phonological processing deficit (Brady and Shankweiler, 1991; Stanovich and Siegel, 1994; Snowling, 2000; Capovilla et al., 2001; Capovilla and Capovilla, 2003). There have been other reports of reading difficulties in the light of preserved phonological awareness skills (Landerl and Wimmer, 2000; de Jong and van der Leij, 2003), leading to the suggestion that poor reading performance might stem from different cognitive impairments. For example, in their study with French-speaking children, Zoubrinetzky et al. (2014) identified four distinct dyslexia subgroups characterized by a single deficit in phonological processing, a single deficit in visual processing, a double deficit, or none of these impairments. Similarly, Wolf and Bowers (1999) proposed that children with dyslexia can present a double deficit in phonological awareness and rapid naming or single deficits with impairments in either phonological awareness or RAN.

A popular subtyping approach has been the distinction between phonological and surface dyslexia. It has been argued that children in the latter group rely extensively on intact phonological strategies for reading and spelling (Coltheart et al., 1983; Castles and Coltheart, 1993). One explanation for our results of impaired reading in the apparent absence of a clear phonological processing deficit could be that children with dyslexia in our study fall into the surface dyslexia subtype. However, children with dyslexia did not show selective impairments in word-level reading skills that would characterize surface dyslexia. Instead, marked difficulties in both word and non-word reading were observed. Furthermore, the classification of children with dyslexia into different subtypes remains controversial. Griffiths and Snowling (2002) concluded that classification systems yield a poor description of the population of individuals with dyslexia at large. Instead, they suggest that the existence of heterogeneous cognitive profiles in dyslexia can be explained by the severity of the phonological deficit, general processing resources, as well as the reading experience of individual children. Indeed, it has now been widely accepted that dyslexia represents the lower end of a continuous distribution of decoding skills in the population (Bishop and Snowling, 2004; Fletcher, 2009; Snowling and Hulme, 2012).

An alternative explanation of our findings is that the instrument that we used to explore explicit phonological processing (PHF) might have been insufficiently sensitive to phonological processing in older children. In that respect, it is critical to note that the authors of the PHF report that the test was developed for the age range from 5 to 10 years (Carvalho et al., 1998), but it was used here with children aged from 7 to 12 years. The measure was used because our study relied on pre-collected data of the Brazilian Dyslexia Association, and the PHF was part of their original assessment battery.

It is clearly established that there is a reciprocal relationship between phonological awareness and learning to read, with the development of reading leading to further refinement of phonological awareness skills (de Jong and van der Leij, 2003; Anthony and Francis, 2005). This has important implications for the assessment of phonological awareness skills in different ages and especially in children who are literate. Whereas measures of phonological awareness in children of preschool age have been repeatedly identified as a valid predictor for early reading achievement and the presence of reading difficulties in the early school grades, phonological awareness assessment often loses its predicate power in older children. In literate children, the best indicator of future reading is often simply reading itself (Landerl and Wimmer, 2000; Bell et al., 2003; Hogan et al., 2005; Rothou et al., 2013). This finding has led some researchers to question the usefulness of assessments of phonological awareness once a certain level of reading has been achieved (Wagner et al., 1997; Torgesen, 1999). As phonological awareness tests lie along a continuum of increasing difficulty, measurements are inaccurate if the specific task demands are not appropriate for a child’s level of development (Vloedgraven and Verhoeven, 2007). In their meta-analytic review, Melby-Lervåg et al. (2012) found that, across orthographies, children with dyslexia presented a stable deficit in phonological awareness between the ages of 5 and 16, if assessed at the level of the phoneme, but effects sizes were reliably smaller if phonological awareness was assessed at the level of the rime. The explicit phonological processing test that was used in our study consisted of a combination of phoneme, rime, and syllable items and also included an articulation task and analyses at the sentence level. Our results concerning explicit phonological processing in dyslexia are likely to be dependent on the way phonological processing was measured. It is possible that if another measure was used (e.g., phonemic awareness), the results might have been different. Further research is needed to explore this issue.

Despite the differences in reading ability, we did not observe significant differences between children with dyslexia and typical readers in most measures of the WISC. Considering our inclusion criteria (children with standard WISC scores above 85 and no comorbid disorders), this finding is not surprising. Previous research has identified different WISC profiles in dyslexia (De Clercq-Quaegebeur et al., 2010). Whereas some studies have shown that children with dyslexia can lag behind their typically developing peers in the verbal, working memory, and processing speed index scores, others have found no such differences (D’Angiulli and Siegel, 2003; Arduini et al., 2006). Inconsistencies in relation to the specific WISC profile in children with dyslexia are likely related to differences in the precise diagnostic criteria for dyslexia across studies. An important characteristic of our group of children with dyslexia and matched controls deserves mention: on the majority of the WISC measures, children’s scores were in the average to high average range. Although there are indications in the data that children with dyslexia performed significantly more poorly than typical readers on some verbal and working memory subtests (VCI—similarities, VCI—information, and WMI—arithmetic), scores on those measures were above the mean scores obtained by the standardization sample representative of the Brazilian population of children (Wechsler, 2013, Brazilian adaptation Rueda et al., 2013). It is possible that by using stringent selection criteria for our dyslexic sample, which can be considered a strength of this study, we minimized the proportion of children with broader oral language or working memory difficulties. Therefore, the children with dyslexia in our study were likely to have a reading deficit that was specific.

Given recent findings indicating that RAN deficits might represent a prime characteristic of reading difficulties (de Jong and van der Leij, 2003; Bicalho and Alves, 2010; Capellini and Lanza, 2010; Georgiou et al., 2012; Germano et al., 2012; Michalick-Triginelli and Cardoso-Martins, 2015; Araújo and Faísca, 2019), we had a specific interest in exploring whether children with a clinical diagnosis of dyslexia in Brazil would present deficits in rapid naming. Findings were straightforward. Children with dyslexia had clear impairments in RAN compared to typical readers. Deficits were found in both alphanumeric and non-alphanumeric RAN tasks, although effect sizes were slightly larger for tasks with alphanumeric stimuli. This result corroborates the findings of a recent meta-analytic review indicating that individuals with dyslexia present large deficits in speeded RAN compared with age-matched typical readers (Araújo and Faísca, 2019). The meta-analysis further showed that the RAN deficit spanned alphanumeric and non-alphanumeric stimulus types and is relatively stable across development (see also Pennington et al., 2001; Clarke et al., 2005). Our study showed the same in readers of Brazilian Portuguese, indicating that RAN deficits cannot simply be explained by differences in letter processing (see also Lervåg and Hulme, 2009). CART analysis showed that RAN was relevant for predicting the likelihood of dyslexia. More specifically, the model developed in this study showed that letter RAN total time, color RAN self-corrections, and color RAN errors predicted dyslexia likelihood with moderate diagnostic accuracy (overall classification accuracy rate of 88.33%, sensitivity of 93.33%, specificity of 83.33%). This finding indicates that RAN abilities are capable of discriminating children with dyslexia from controls. Similar conclusions were obtained in other studies with readers of Brazilian Portuguese showing that RAN is a valid tool for the identification of individuals at risk for reading difficulties (Justi and Cunha, 2016), as well as for children and adolescents with reading impairments (Lima et al., 2008; Capellini and Conrado, 2009; Bicalho and Alves, 2010; Capellini and Lanza, 2010; Germano et al., 2012; Silva et al., 2012; Michalick-Triginelli and Cardoso-Martins, 2015). As Portuguese is considered an intermediate orthography in terms of regularity, these findings give support to the position that RAN deficits might not be affected by the transparency of the writing system (Araújo and Faísca, 2019; Landerl et al., 2019).

The results from this study should be interpreted with caution. The study comprised a small sample of children, and sensitivity and specificity were not investigated in an independent sample. While CART analysis is a valid classification tool, it is prone to overfitting the data for small samples (Li and Belford, 2002). Small variations in the data might therefore result in a different decision tree or cutoff points. Cross-validation showed that overfitting was indeed a problematic issue, suggesting that the generalizability of our model is limited. Furthermore, as discussed before, problems with the measurement of explicit phonological processing make it difficult to draw strong conclusions in relation to phonological awareness skills in dyslexia, and it would be premature to conclude that children in our study presented a single deficit in RAN. It is possible that our study underestimates the degree to which phonological awareness is impaired in developmental dyslexia. Further studies are needed, including different measurements of phonological awareness and RAN, to explore the double deficit account of dyslexia (Wolf and Bowers, 1999; Cronin, 2011; Asadi and Shany, 2018). Another limitation of this research is that almost all the children in the study came from middle- to high-income families (that can afford Brazilian private school education). Clearly, further studies are needed to assess the generalizability of our findings to a larger and more representative sample of Brazilian children. In case our preliminary results are confirmed, it will be important to investigate the cognitive factors behind RAN. As mentioned in the *Introduction*, there is still no consensus regarding the underlying processes of RAN, how they contribute to the development of reading, or whether intervention approaches targeting RAN might lead to improvements in reading (Lervåg and Hulme, 2009; Araújo and Faísca, 2019; Clayton et al., 2020).

Taking these together, this study from Brazil suggests that dyslexia is associated with RAN impairments in children who are learning to read in Portuguese. More specifically, children with poor performance on letter RAN were more likely to have a diagnosis of developmental dyslexia. These results indicate that RAN is predictive of dyslexia diagnosis likelihood and might represent a relevant clinical marker of dyslexia in children from Brazil.

## Data Availability Statement

All datasets generated for this study are included in the article/supplementary material.

## Ethics Statement

The studies involving human participants were reviewed and approved by the Mackenzie Presbyterian University Research Ethics Committee. Written informed consent to participate in this study was provided by the participants’ legal guardian/next of kin.

## Author Contributions

PS designed the study, collected and analyzed the data, and wrote the manuscript. PE contributed to the interpretation of the data and wrote, revised, and refined the manuscript. PL analyzed the data and contributed to the writing of the manuscript. MN and LS contributed to data collection. RT contributed to the writing of the manuscript. EM designed the study, analyzed the data, and contributed to the writing of the manuscript. All authors provided critical feedback.

## Conflict of Interest

PS and EM declare that they have a commercial interest in a RAN standardized test called TENA (Teste de Nomeao Automtica, Silva et al., 2018). The TENA test was not used in this research manuscript. The remaining authors declare that the research was conducted in the absence of any commercial or financial relationships that could be construed as a potential conflict of interest.
